# A Tangible Educative 3D Printed Atlas of the Rat Brain

**DOI:** 10.3390/ma11091531

**Published:** 2018-08-25

**Authors:** Darío R. Quiñones, Jorge Ferragud-Agulló, Ricardo Pérez-Feito, Juan A. García-Manrique, Santiago Canals, David Moratal

**Affiliations:** 1 Centre for Biomaterials and Tissue Engineering, Universitat Politècnica de València, 46022 Valencia, Spain; dariomrxpro2@hotmail.com (D.R.Q.); jorgeferragudagullo@gmail.com (J.F.-A.); 2 Thermodynamics Department, Universitat Politècnica de València, 46022 Valencia, Spain; riperez@upvnet.upv.es; 3 Institute of Design for Manufacturing and Automated Production, Universitat Politècnica de València, 46022 Valencia, Spain; jugarcia@mcm.upv.es; 4 Instituto de Neurociencias, Consejo Superior de Investigaciones Científicas—Universidad Miguel Hernández, 03550 Sant Joan d’Alacant, Spain; scanals@umh.es

**Keywords:** brain, rapid prototyping, atlas, rat, magnetic resonance imaging, educative model

## Abstract

In biology and neuroscience courses, brain anatomy is usually explained using Magnetic Resonance (MR) images or histological sections of different orientations. These can show the most important macroscopic areas in an animals’ brain. However, this method is neither dynamic nor intuitive. In this work, an anatomical 3D printed rat brain with educative purposes is presented. Hand manipulation of the structure, facilitated by the scale up of its dimensions, and the ability to dismantle the *“brain”* into some of its constituent parts, facilitates the understanding of the 3D organization of the nervous system. This is an alternative method for teaching students in general and biologists in particular the rat brain anatomy. The 3D printed rat brain has been developed with eight parts, which correspond to the most important divisions of the brain. Each part has been fitted with interconnections, facilitating assembling and disassembling as required. These solid parts were smoothed out, modified and manufactured through 3D printing techniques with poly(lactic acid) (PLA). This work presents a methodology that could be expanded to almost any field of clinical and pre-clinical research, and moreover it avoids the need for dissecting animals to teach brain anatomy.

## 1. Introduction

The use of animals for scientific or educational purposes presents a great moral dilemma for current society. Although social progress leads to greater empathy towards animals, it is true that experimentation with them has allowed a large advance in the neuroscience field. This type of experimentation has helped the study of brain diseases and their causes, which would not have been possible otherwise according to current knowledge. However, nowadays, it is possible to study the brain without harming animals thanks to neuroimaging technologies.

Neuroimaging technologies, such as Computed Tomography (CT) and Magnetic Resonance Imaging (MRI), are indispensable tools for the diagnosis and treatment of central nervous system diseases [1,2,3,4,5,6]. Even though MRI and CT techniques collect three-dimensional (3D) data, clinicians typically view these data on two-dimensional (2D) screens. 2D representations limit our appreciation of complex structures, such as brain convolutions, and 2D displays of these models on computer screens prevent us from interacting with a model of the physical brain. Furthermore, appreciating complex 3D spatial relationships in a 2D screen requires a strong foundational understanding of anatomy and mental 3D visualization skills [7].

A tangible physical model based on MR images or digital templates would permit biologists, neuroscientists and students to complement information obtained by 2D and virtual 3D imaging. Furthermore, physical 3D models could be more advantageous for clinical education than computer-based 3D models [7,8,9]. Therefore, high-resolution multiscale and multimodal 3D models of the brain can be seen as essential tools to understand its complex structural and organization [10]. A commercial rat brain model called “C29 Rat Brain Comparative Anatomy” (3B Scientific GmbH, Hamburg, Germany) exists for educational purposes, but it is not detailed enough for scientific purposes.

The most widely used MRI template of a rat brain is that developed by Schwarz et al., [11]. It is co-registered with the stereotaxic co-ordinates of the Paxinos and Watson (1982) digital atlas [12]. The template set provides anatomical images as well as tissue class probability maps for brain parenchyma and CerebroSpinal Fluid (CSF). The use of this template paves the way for the use of standard fMRI software for tissue segmentation of rat brain data. This enables the atlas structure and the stereotaxic coordinates that correspond to a feature within a statistical map to be interactively reported, which facilitates the localization of functional effects. Furthermore, voxels, which are within selected brain structures, can be combined to define anatomically based 3D volumes of interest (VOIs) [11]. The use of standard functional Magnetic Resonance Imaging (fMRI) software for tissue segmentation of rat brain data is facilitated by the use of this template.

3D printing is rapidly becoming a source of novel biomedical applications, as well as a cost-effective mean of producing customized 3D objects [13,14,15,16], for example, printing organic tissue [17] or developing scaffolds for tissue engineering [18]. However, some of the most complex applications use high-end industrial printers that are too expensive for educative purposes or even for research centers with limited funds. Nonetheless, the progression of open source platforms has increased the capabilities and popularity of desktop 3D printers.

In this work, we describe a step-by-step, end-to-end easily replicable methodology to generate and scale a 3D printed model of a rat brain with the most important macroscopic regions. It is based on an MRI template of the rat brain co-localized with the Paxinos and Watson rat brain atlas, combined with 3D computer-aided design (CAD) tools and 3D prototyping technologies to obtain a didactic 3D printed brain. Furthermore, each 3D printed region of the brain is designed to be easily attached to or detached from the other parts. This model could have a big impact on the way biologists and neuroscientists teach in the future. It makes more “tangible” the understanding of the relevant areas of the brain for teaching, while reduces the use of experimental animals for these purposes. In addition, the methodology can be extrapolated to different regions of the brain or even to other body regions.

## 2. Materials and Methods

**Figure 1:**
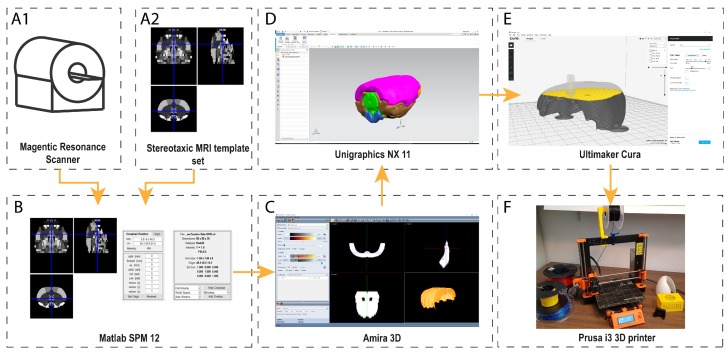
This figure shows the pipeline of the process from a CT or MRI set of images to a physical 3D printed model. First, images from a CT or MRI scanner (**A1**) are colocalized with a stereotaxic template (**A2**). Next, these images are clustered in Matlab SPM (**B**) to create the Voxels of Interest (VOI). With this set of images, a 3D reconstruction is created in Amira 3D software (**C**). Then, the 3D surface, which was created in Amira, is opened in a 3D design tool, e.g. Unigraphics NX, (**D**) where 3D surfaces are converted into 3D solid bodies. These solid bodies are provided with joins and connectors to add detachable properties to the 3D printed body. Once this process is complete, the manufacturing process starts (**E**). This consists of transforming the 3D body into a physical set of coordinates, which the 3D printer can interpret. In this case, a Prusa i3 MK2 3D printer with Cura software was used (**F**).

The methodology presented in this paper consists of the following steps to generate 3D objects from clinical or pre-clinical: (i) selection of the data; (ii) segmentation of the region of interest (ROI); (iii) transformation of the data from 2D ROIs to a 3D triangular mesh; (iv) editing the 3D mesh (to clean uneven areas, add support structures, etc.); (v) transferring the data to 3D printer software in order to create printing coordinates; and (vi) sending it to production (Figure 1).

### 2.1. Data Selection: The Eight Most Relevant Macroscopic Areas in the Rat Brain

The first step in creating 3D printed models from a digital atlas is the selection of the regions of interest. The image data choice is important; low-resolution images can result in a discrepancy between the generated model and actual anatomy. In this particular case, a stereotaxic MRI template set for the rat brain with tissue class distribution maps made by Schwarz et al., [11] and co-registered anatomical atlas “*The Rat Brain in Stereotaxic Coordinates*” from *Paxinos and Watson (1982)* [12], was chosen. This document shows sagittal and coronal cuts throughout the brain of a specimen rat. The atlas partially contains all the encephalon of a rat, but the brain is the main part which appears in the MRI atlas. However, the whole cerebellum, the medulla and the olfactory bulb are not included in the atlas.

The MRI template that was used is the one presented in [11]. Briefly, this template was created from multi-slice T2-weighted images of a rat brain acquired using a RARE sequence on a Bruker Avance 4.7-T MR scanner (Billerica, MA, USA) with the following acquisition parameters: RARE factor 8, matrix 256 × 256; FOV 40 mm, slice thickness 1 mm, 32 contiguous coronal slices; TReff = 5500 ms; TEeff = 76 ms. A 72-mm birdcage resonator was used for RF transmission and the Bruker quadrature FRat Brain_ coil was used for RF reception. The images were acquired in the coronal plane, centered 8 mm caudal from the posterior edge of the olfactory bulb [11]. Each region is marked with a different gray intensity. In total, 466 brain regions were clustered into 38 sub-regions and grouped into eight regions with the participation of an experienced neurobiologist. These parts correspond to the eight pieces of the printed rat brain that is presented in this paper (Table 1).

**Figure 2:**
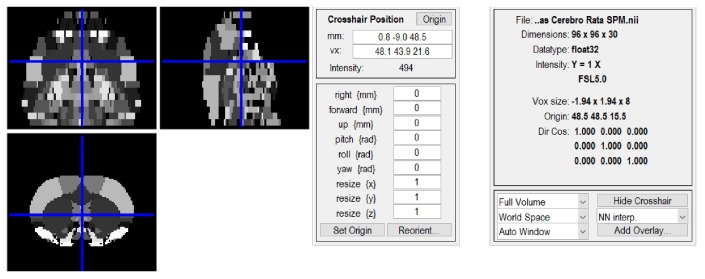
Statistical Parametrical Mapping toolbox. The image represents a slice of the employed digital atlas, whereas the specific coordinates are shown in SPM 12.

To visualize and choose the desired areas from the stereotaxic MRI template set, a specific software was needed. In this case, Statistical Parametric Mapping 12 (SPM12) [19], which is designed as a MATLAB (The Mathworks Inc., Natick, MA, USA) toolbox, was chosen (Figure 2), because it is the most suitable and open source software available for the this purpose [20,21]. SPM is an academic software toolkit for the analysis of functional imaging data for users familiar with the underlying statistical, mathematical and image processing concepts. SPM12 can be also executed on GNU/Octave [22], which is an Open-Source alternative to Matlab. Once the areas were chosen in the atlas and identified in the MRI template set, they were grouped in SPM and saved in different files in *NIFTI* format (.nii).

### 2.2. Three-Dimensional Reconstruction of the Desired Areas

Once all the macroscopic areas were chosen, a way to represent them in a 3D view was needed. Amira^TM^ (Thermo Scientific^TM^, Waltham, MA, USA) software was selected for 3D visualization and analysis. Amira is a powerful software tool capable of dealing with life science data from computed tomography, microscopy, MRI, and many other imaging modalities. Mango (UT Health, Houston, TX, USA) is an Open-Source alternative to Amira that can be use to the same purpouse.

**Figure 3:**
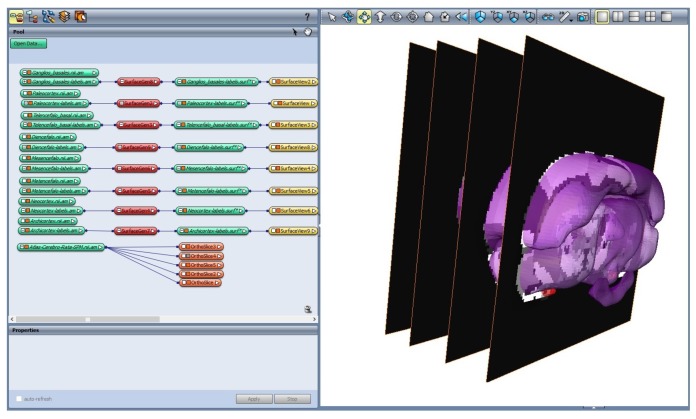
Data labeling in Amira 3D software. A set of slices is displayed, which belong to the Neocortex region, from the selected dataset. This functionality selects in each of the slices the regions with the same gray level.

Figure 3 shows the chosen regions using Amira. Through the functionality “*Labeling*”, it was possible to group all the Regions of Interest (ROI) from each slice of the dataset. Once the labeling process was complete, and using the functionality “*SurfaceGen*”, all the labeled areas on each slice were transformed into a surfaced 3D body (facet body).

This body was exported as an *OBJ* file, which is an extended open source format. The *OBJ* file format is a simple data-format that represents 3D geometry. It gives the position of each vertex, the vertex normals, and the faces that make each polygon defined as a list of vertices, and texture vertices. However, this format has a drawback: it is not easily editable because it only represents the surface of the 3D body with the necessary number of triangles. It does not represent the 3D object as 3D solid body. This means that Boolean 3D operations (e.g., add, subtract, and intersect) cannot be easily applied. In our specific case, it was necessary to edit the geometry of the resulting 3D facetted body, because the objective was to create an easily detachable 3D brain.

### 2.3. Transforming the Facet Body into an Editable Solid Body

**Figure 4:**
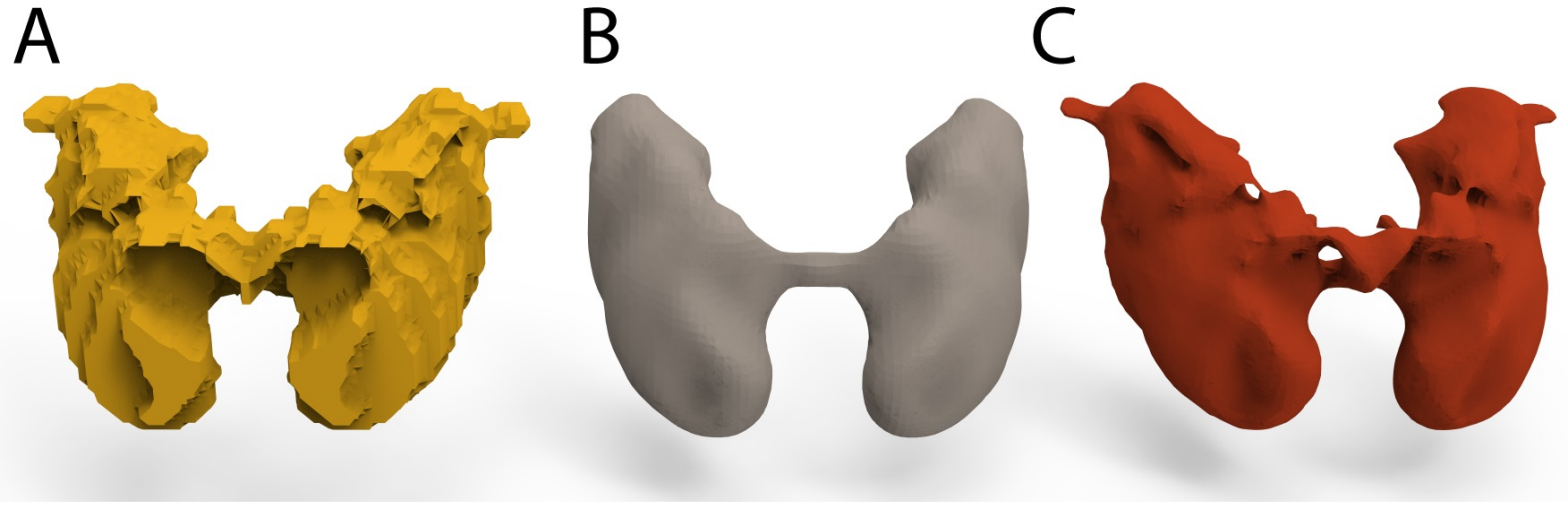
Smoothing process applied on the Basal Ganglia: (**A**) Basal ganglia before being smoothed; (**B**) smoothing process with Amira 3D software; and (**C**) smoothing process with Unigraphics NX.

To create a 3D brain with interlocking plastic parts, it was necessary to edit the facet bodies that were previously generated, and then to add fastener joins. This process was performed with Unigraphics NX 11 (Siemens, Munich, Germany). This software, widely used in the industry, enables design, structural analysis, simulations and image study, among other things. Through a program of this type, it is possible to create 2D and 3D designs from scratch, or to modify files from other sources. FreeCAD (Blender Foundation, Amsterdam, The Netherlands) can be used as Open-Source alternative to Unigraphics NX. Using these features, the OBJ files were imported and analyzed as a convergent body. Then, each analyzed part was converted into an editable 3D solid body. However, as shown in Figure 4A, the resulting shape is not as smooth as could be expected. These uneven surfaces are due to the low resolution of the template. To correct this, a constrained smoothing process was applied to each of the eight macroscopic regions. There are two methods of smoothing the surface of the 3D object. One, which is less computationally demanding, is using Amira 3D, but it has some drawbacks, as shown in Figure 4B. This method creates a well-smoothed body, but it suppresses relevant details of the region.

The other method is more computationally demanding and involves a tool of Unigraphics NX called “*surface smoothing*”, which allows smoothing of the surfaces without losing the relevant morphology of the region. This second method was chosen for all bodies that were extracted from the template.

To create the joins for each component, the ideal position of the join was studied for each part. According to the part dimension, the position of the join was determined as an extrusion (male) or a depression (female). Then, each join was added to the corresponding part in Unigraphics NX by converting the join and the part into a unique 3D solid body.

**Figure 5:**
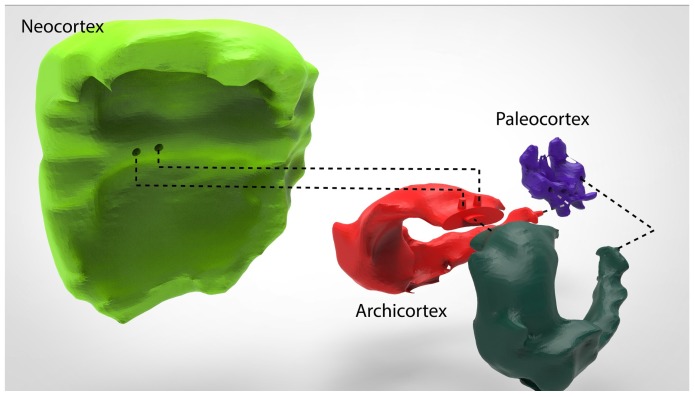
Interconnecting joins. Figure shows the interconnecting joins among three parts of the brain 3D model. It is possible to appreciate that the Neocortex, as well as the Paleocortex, has two depressions. These depressions fit perfectly with the protuberances on the top of the Archicortex.

The joins were added with a 5% tolerance to facilitate attach and detach parts after being 3D printed. As shown in Figure 5, each join was created in a specific place and as specific shape to facilitate an interconnection between the parts. The Archicortex was further divided into two parts to make it easy to assemble and disassemble the inner parts of the brain.

### 2.4. Rapid Prototyping

Rapid prototyping can be defined as an approach or methodology used to quickly manufacture physical models using 3D CAD data. Rapid prototyping has also been referred to as solid free form, computer-automated or layered manufacturing. Rapid prototyping has its obvious use as a truly 3D method for visualization and better haptic interaction.

Nowadays, 3D home printers have become more accessible than industrial 3D printers, and this has increased the number of “Do It Yourself” (DIY) projects that are underway. The most affordable and common type of rapid prototyping is Fused Deposition Modelling (FDM) technology, also known as Fused Filament Fabrication (FFF). There are other types of rapid prototyping, such as Stereolithography (SLA) or Selective Laser Sintering (SLS) [23], which are more accurate and detailed but which cost more [24]. FDM technology prints an object one layer at a time by extruding heated plastic through a nozzle, while moving around a flat hot surface (hot bed).

A Prusa i3 MK2 with a precision of 50 microns per layer height, and a printing surface of 10,500 cm^3^ (25 × 21 × 20 cm or 9.84 × 8.3 × 8 in) was used to print all of the parts. With these manufacturing constraints and the modified solid models with the joins, a scale of four times the actual size (4:1) was chosen in the printing software Ultimaker Cura (Ultimaker B.V., Geldermalsen, The Netherlands).

## 3. Results

**Figure 6:**
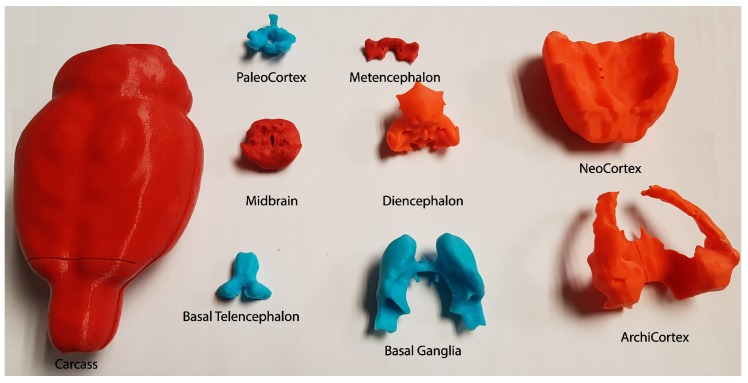
Final 3D model (disassembled) of the rat template.

The final 3D printed model of the rat brain (Figure 6) is composed of the eight smoothed parts, all parts of which fit together perfectly. The scaled measures of each part are shown in Table 2. In addition, an external container was made to accommodate the eight regions.

**Figure 7:**
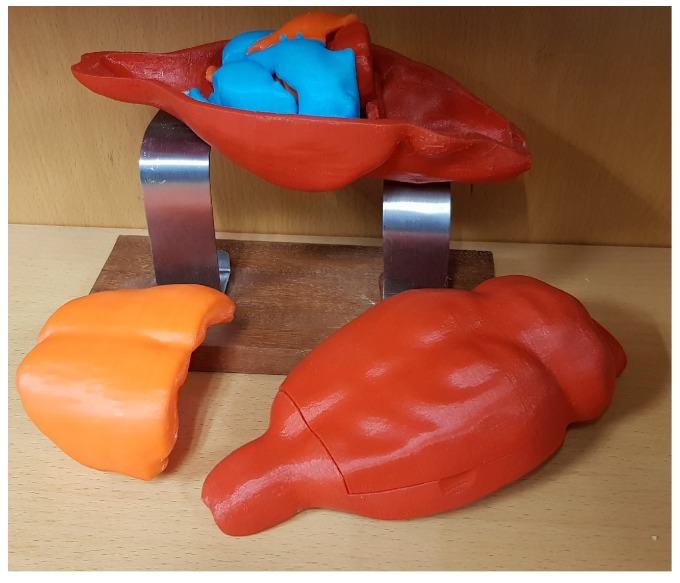
Final 3D model of the rat template.

This container was designed using the external contour of the brain in the template. It was then enlarged a little (4.2:1) to facilitate the accommodation of the other parts. In addition, the cover was divided in two to make it possible to open, as shown in Figure 7. Figure 6 shows the whole-brain model disassembled, all eight parts and the container that encloses them. Figure 7 shows that all of the parts fits perfectly together.

In terms of material for the printing process, poly(lactic acid) (PLA) was chosen. This material is a thermoplastic polymer that is made from renewable resources. It melts at 160 degrees Celsius and it is less rigid than Acrylonitrile Butadiene Styrene (ABS), another commonly used material in 3D printing. This is important for the 3D model because it makes the printed parts more elastic and shock-resistant.

## 4. Discussion

Rapid prototyping has grown beyond its initial use in industrial sectors, e.g., the automobile industry, and today can be regarded as one of the most promising techniques for use in medical imaging. Although medical applications are relatively recent, their enormous potential has already been demonstrated in several studies [25,26,27]. Rapid prototyping objects are useful for training surgeons, as they allow surgical procedures to be simulated in a realistic manner. Even though medical research has already benefitted from rapid prototyping [28,29] and efforts have been made in the development of artificial organs and tissues [30,31], the traditional approach of teaching anatomy remains the same. This approach focuses mainly on MRI, CT and dissections of reals animals, without taking into consideration any variations and pathological changes. Currently, there is a well-known rat brain model called “C29 Rat Brain Comparative Anatomy”, which aims for educative point of view, but is not detailed enough for scientific purposes.

The 3D printed brain that is presented in this paper could serve as the medium for bringing anatomical variations to pre-clinical studies in order to improve the understanding of anatomy [7] while preserving animal lives. In addition, by using other materials to adapt transparency or rigidity, certain aspects can be emphasized for the trainee and the student.

In the scientific domain, adding the cranium of the subject to the 3D model, we envision applications of this approach in designing and probing brain implant prototypes, as recording chambers, electric and/or optogenetic stimulation holders, and designing coils for transcranial magnetic or direct current stimulation.

## 5. Conclusions

In this paper, a step-by-step, end-to-end easily replicable methodology to generate a 3D printed model of a rat brain has been presented. It is based on an MRI template of the rat brain co-localized with the Paxinos and Watson rat brain atlas, combined with 3D CAD tools and 3D prototyping technologies to obtain a tangible 3D printed atlas of the rat brain. In addition, each 3D printed region is designed to be easily attached or detached from the other parts. This model could have a big impact on the way biologists and neuroscientists teach in the future. It makes more “tangible” the understanding of the relevant areas of the rat brain for teaching, while reduces the use of experimental animals for these purposes. In addition, the methodology can be extrapolated to different regions of the brain or even to other body regions.

To obtain the necessary images to generate the 3D parts, a digital atlas, which was correlated with a stereotaxic anatomical atlas, was used. With the participation of an experienced biologist, 466 structures were extracted and clustered into eight different parts, which correspond to the eight pieces of the printed rat brain that is presented in this paper. These parts are the Neocortex, the Archicortex, the Paleocortex, the Basal Ganglia, the Basal Telencephalon, the Diencephalon, the Mesencephalon, and the Metencephalons. Each part has joins to fasten together the different parts. In addition, a housing formed by three additional pieces was created. This in turn performs the function of storing the rest of the pieces and serves as a spatial orientation for the assembly of the brain. In further research, a complete rat brain with 38 sub-regions will be developed.

This work presents a methodology that could be expanded to almost any field of clinical and pre-clinical research, and moreover it avoids the need of dissect animals to teach anatomy. In addition, all parts are available at http://dmoratal.webs.upv.es/research.html.

## Figures and Tables

**Table 1:** Regions clustering.

Regions	Sub-Regions
Neocortex	Cortex Parietal Association, Cortex Retrosplenial, Cortex Temporal Association, Corpus Cortex Sensory, Cortex Motor, Cortex Insular, Cortex Frontal Association, Cortex Orbitofrontal, Cortex Medial Prefrontal, Cortex Cingulate, Collosum
Archicortex	Hippocampus, Cortex Entorhinal Piriform
Paleocortex	Olfactory Nuclei, Olfactory Tubercle
Basal Ganglia	Globus Pallidus, Amygdala, Diagonal Band, IPAC, BNST, Caudate Putamen, Ventral Pallidum, Substantia Innominata, Caudate Putamen
Basal telencephalon	Septum, Accumbens
Diencephalon	Hypothalamus, Thalamus, Medial Geniculate, Zona Incerta
Midbrain	Raphe, Mesencephalic Region, Substantia Nigra, Periaqueductal Grey, Superior Colliculus, Interpeduncular Nucleus, VTA (Ventral Tegmental Area)
Metencephalon	Pons

**Table 2:** Measurements on X, Y, and Z axes of each part, total assembly without container and total assembly with container.

Part	X (mm)	Y (mm)	Z (mm)	Part	X (mm)	Y (mm)	Z (mm)
Neocortex	59.60	28.94	54.40	Midbrain	26.72	24.90	15.10
Archicortex1	28.58	30.19	50.38	Metencephalon	26.43	12.48	6.02
Archicortex2	32.08	30.19	50.38	Bottom Cover	69.54	30.08	131.73
Paleocortex	24.00	17.94	18.69	Upper Cover	69.07	39.04	132.66
Basal ganglia	51.01	25.98	34.83	Small Cover	64.46	13.73	49.60
Basal Telencephalon	22.83	18.45	15.84	Total without cover	59.60	38.37	57.60
Diencephalon	33.02	24.35	30.35	Total with cover	69.54	52.34	132.66

## Abbreviations

The following abbreviations are used in this manuscript:

NIFTI	Neuroimaging Informatics Technology Initiative
OBJ	File format is a simple data-format that represents 3D geometry alone
CAD	Computer-Aided Design
FDM	Fused Deposit Modeling
FFF	Fused Filament Fabrication
SLS	Selective Laser Sintering
SLA	Stereolithography
